# Diverging landscape impacts on macronutrient status despite overlapping diets in managed (*Apis mellifera*) and native (*Melissodes desponsa*) bees

**DOI:** 10.1093/conphys/coaa109

**Published:** 2020-12-15

**Authors:** Christina L Mogren, María-Soledad Benítez, Kevin McCarter, Frédéric Boyer, Jonathan G Lundgren

**Affiliations:** 1 Department of Plant and Environmental Protection Sciences, University of Hawai’i at Mānoa, 3050 Maile Way Gilmore 310, Honolulu, HI 96822, USA; 2 Ohio Agricultural Research and Development Center, The Ohio State University, Wooster, OH 44691, USA; 3 Department of Experimental Statistics, Louisiana State University, Baton Rouge, LA 70802, USA; 4 Laboratoire d’Écologie Alpine, Centre National de la Recherche Scientifique, Université Grenoble Alpes, F-38000 Grenoble, France; 5 Ecdysis Foundation, Estelline, SD 57234, USA

**Keywords:** Apidae, conservation physiology, dietary composition, honey bees, metabarcoding, nutrition

## Abstract

Declining pollinator populations worldwide are attributed to multiple stressors, including the loss of quality forage. Habitat management in agricultural areas often targets honey bees (*Apis mellifera* L.) specifically, with the assumption that native bees will benefit from an ‘umbrella species’ strategy. We tested this theory using a conservation physiology approach to compare the effects of landscape composition and floral dietary composition on the physiological status of honey bees and *Melissodes desponsa* in eastern South Dakota, USA. The total glycogen, lipid and protein concentrations were quantified from field collected bees. Next-generation sequencing of the *trn*L chloroplast gene from bee guts was used to evaluate dietary composition. The effects of landscape and dietary composition on macronutrient concentrations were compared between bee species. As the mean land-use patch area increased, honey bee glycogen levels increased, though *M. desponsa* experienced a decrease in glycogen. Protein levels decreased in honey bees as the largest patch index, a measure of single patch dominance, increased versus *M. desponsa*. Lipids in both species were unaffected by the measured landscape variables. Dietary analysis revealed that honey bees foraged preferentially on weedy non-native plant species, while *M. desponsa* sought out native and rarer species, in addition to utilizing non-native plants. Both species foraged on Asteraceae, Oleaceae and Fabaceae, specifically *Melilotus* sp. and *Medicago* sp. Dietary composition was not predictive of the macronutrients measured for either species. Together, these data highlight the management importance of including patch area in conservation recommendations, as bee species may have divergent physiological responses to landscape characteristics. While solitary bees may forage on weedy introduced plants in agricultural areas, robust strategies should also reincorporate native plant species, though they may not be preferred by honey bees, to maximize overall health and diversity of pollinator communities.

## Teaser

We investigated the roles of diet and landscape diversity on the health of two bee species. Both shared many of the same food resources. However, physiological responses to landscape changes were significantly different. Conservation strategies should focus on the size of pollinator plantings and flower diversity to improve bee health.

## Introduction

Habitat conversions at the landscape scale can negatively affect pollinator diversity by restricting available resources to those that promote agrobionts, or species thriving in areas heavily modified for agricultural production (Naug, 2009; Mogren *et al.*, 2016). In the Northern Great Plains of the Midwestern USA, prairie and grassland conversions to agricultural monocultures have greatly simplified the landscape of eastern South Dakota (Wright and Wimberly, 2013; Fausti, 2015; Wimberly *et al.*, 2017; Otto *et al.*, 2018). The additional removal of historic tree stands and flowering vegetation at field margins to maximize the field size and control weeds has further limited available floral resources upon which a large number of native bees rely (Mogren *et al.*, 2016). In these types of intensively managed agricultural areas, lands set aside for conservation have proven particularly important in maintaining pollination services of managed bees such as honey bees (*Apis mellifera* L.) (Gallant *et al.*, 2014; Otto *et al.*, 2016; Smart *et al.*, 2016) and native bees (Benjamin *et al.*, 2014), although the effects may vary among cropping systems (Campbell *et al.*, 2017; Quinn *et al.*, 2017). For this reason, the Pollinator Health Task Force (2015) recommended South Dakota along with Michigan, Minnesota, Montana, North Dakota and Wisconsin for a pollinator conservation initiative incentivizing habitat restoration. This focus on habitat restoration in agricultural areas is often intended to promote honey bees specifically, with an assumption that these conservation efforts will benefit native bees and other pollinators through an umbrella species paradigm (Kalinkat *et al.*, 2017).

However, significant differences between resource utilization exist between honey bees, which are social, and native bees, which are mostly solitary (e.g. Steffan-Dewenter and Tscharntke, 2000). Honey bees have a high forage fidelity (Free, 1963) combined with the ability to mass recruit to plentiful resources and thus would be expected to forage mostly upon mass blooming resources. In contrast, solitary bees may visit numerous floral species in a single foraging visit. As such, floral management considerations for one type of pollinating bee may not completely satisfy the needs of the other.

One means for identifying differences between the diets of eusocial and solitary bees is through the use of next-generation sequencing analysis. DNA metabarcoding to identify dietary diversity in a number of animal species, including honey bees (Hawkins *et al.*, 2015; Smart *et al.*, 2017) is a technique that can be employed to compare the diets of coexisting species. DNA metabarcoding also results in greater taxonomic resolution than palynology alone (Keller *et al.*, 2015; Richardson *et al.*, 2015; Valentini *et al.*, 2010). In gut-content analysis applications, the chloroplast *trn*L (UAA) p6 intron region (Taberlet *et al.*, 1991) has been used to successfully categorize the diets of herbivorous mammals (Hibert *et al.*, 2013; Srivathsan *et al.*, 2015; Gebremedhin *et al.*, 2016; Espunyes *et al.*, 2019) and invertebrates (Valentini *et al.*, 2009; Wallinger *et al.*, 2015; Sint *et al.*, 2018). This gene region is highly conserved yet generally small enough that it may more easily be detected in degraded DNA samples as would be encountered in partially digested plant materials in herbivore guts (Taberlet *et al.*, 2007).

Discerning cause-and-effect relationships between the physiology of an organism and the environment in which they reside in order to refine resource management strategies to benefit that species is an emerging area of study, termed conservation physiology (Cooke *et al.*, 2013). This has important implications for management of pollinators (Alaux *et al.*, 2017). Assessment of health and nutrient parameters in field-collected bees adds an important, *in situ* perspective into the effects of habitat manipulations on their physiological status, as nutritional stress is associated with reductions in learning (Jaumann *et al.*, 2013), foraging ability (Scofield and Mattila, 2016) and immunity (Alaux *et al.*, 2010; Dolezal *et al.*, 2016). Floral resource availability (Di Pasquale *et al.*, 2016; Smith *et al.*, 2016) and landscape diversity (Dolezal *et al.*, 2016; Alaux *et al.*, 2017) have been previously correlated with bee physiology measures separately. However, they are not often considered simultaneously.

The purpose of this study was to define how landscape and plant diversity within a highly developed agricultural area impact the nutritional status of honey bees and a ubiquitous solitary native bee, *Melissodes desponsa* (Apidae, Eucerini). We tested the hypothesis that a more diverse agricultural landscape and access to more diverse forage resources would contribute to greater overall health in these bee species as measured by lipid, glycogen and protein macronutrients. By comparing these physiological measurements of a managed and native bee coexisting in the same region, we aim to translate these results into proactive management strategies for a region identified as important for pollinator conservation.

## Materials and methods

### Study region and bee collection

**Figure 1:**
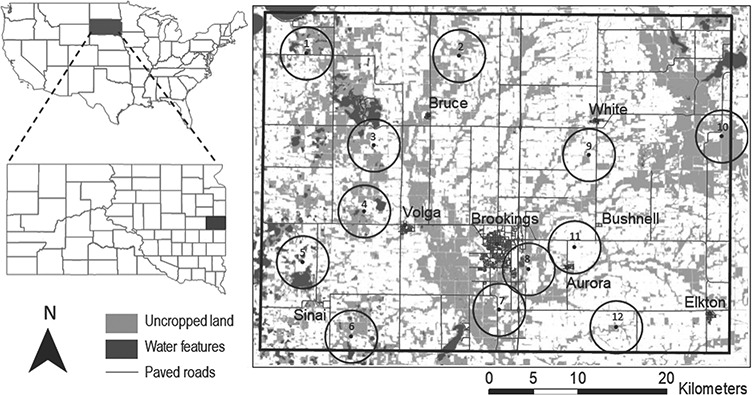
Map of the study region, showing 3 km sampling buffers for the landscape analysis. Figure originally published in Environmental Entomology (Mogren *et al.*, 2016).

Bees were collected from 12 locations throughout Brookings County in east-central South Dakota in August of 2013 (Fig. 1), which reflected a gradient of landscape-level diversity conditions: within a 3-km radius of collection locations, row crops accounted for 26.3–77.6% of the landscape; grass and pasture, 8.1–65.1%; forage crops, 4.0–30.2%; small grains, 0–4.1%; and aquatic habitat, 0.2–25.4% (Mogren *et al.*, 2016). Precipitation totals fell below the historical average of 78 mm, while temperatures during bee collection ranged from 26.1°C to 33.3°C.

During the bee diversity assessment reported in Mogren *et al.* (2016), *M. desponsa* (Apidae: Eucerini) was the most abundant native bee species recovered from bee bowls and blue vane traps (SpringStar Inc., Woodinville, WA, USA) across collection sites and thus were chosen as a model solitary bee species. These traps were deployed along crop field margins. Honey bee foragers were collected from apiaries located adjacent to each of the collection locations. Although the life histories for these bee species differ (e.g. honey bee foragers that are non-reproductive versus foraging *M. desponsa* females that are reproductive), comparing females actively engaged in foraging behaviours normalizes these differences. Samples were preserved in 70% ethanol and transported back to the laboratory on ice. Female *M. desponsa* were sorted and identified from the pollinator survey samples. All bees used in the present study were stored at −80°C until analysis. The number of bees of each species analysed per site is presented in Table 1.

**Table 1:** Summary of health metrics (mean ± SE), total number of bees analysed (N) and number of bees testing positive for *trn*L by sampling site

**Site**	**Honey bees**					***M. desponsa***				
	**N**	***trn*L+**	**Glycogen^a^**	**Lipids**	**Protein**	**N**	***trn*L+**	**Glycogen**	**Lipids**	**Protein**
1	10	8	43.2 ± 4.55	63.8 ± 12.5	13.0 ± 1.28	6	6	35.5 ± 2.10	94.4 ± 26.1	11.8 ± 3.19
2	10	7	50.5 ± 12.8	43.2 ± 10.4	12.3 ± 1.33	6	4	32.5 ± 8.45	57.5 ± 14.0	8.32 ± 1.00
3	10	9	51.6 ± 12.1	83.3 ± 14.4	11.5 ± 0.91	5	5	25.5 ± 2.81	44.6 ± 10.1	11.3 ± 1.95
4	10	9	54.4 ± 10.5	61.4 ± 8.53	8.77 ± 0.73	8	7	32.9 ± 3.41	42.7 ± 13.7	10.9 ± 1.10
5	10	9	32.1 ± 5.45	52.4 ± 13.6	11.6 ± 1.04	4	4	45.5 ± 10.3	79.5 ± 10.6	8.30 ± 0.72
6	10	8	47.3 ± 9.56	61.2 ± 15.4	10.2 ± 1.15	7	7	24.8 ± 6.25	50.6 ± 8.55	9.36 ± 0.65
7	10	9	53.0 ± 10.2	54.4 ± 11.2	11.9 ± 1.16	5	4	32.6 ± 6.67	70.0 ± 14.9	9.81 ± 0.68
8	10	9	49.6 ± 11.5	58.9 ± 14.0	10.7 ± 1.26	6	6	33.8 ± 5.27	47.5 ± 15.9	9.37 ± 1.20
9	10	8	42.5 ± 6.01	29.9 ± 5.03	10.3 ± 1.29	2	2	42.5 ± 5.26	56.5 ± 16.9	9.49 ± 2.29
10	10	10	47.8 ± 6.45	60.7 ± 13.0	15.1 ± 1.59	4	4	64.2 ± 42.0	32.2 ± 15.3	8.29 ± 1.97
11	10	8	48.4 ± 5.58	51.8 ± 12.6	8.36 ± 1.48	17	16	48.1 ± 10.2	71.1 ± 14.5	13.6 ± 0.92
12	10	9	39.4 ± 4.21	29.7 ± 6.61	10.1 ± 1.73	3	2	32.1 ± 5.16	18.6 ± 4.87	11.2 ± 1.34

^a^ Units are μg/bee for glycogen and lipids and mg/bee for protein.

### Macronutrient assays

Bee nutrient analyses for lipids, glycogen and protein were conducted following Mogren and Lundgren (2016) and used as relative proxies for bee health. Briefly, bees were rinsed in 10% bleach and water. The entire alimentary canal was removed and stored in 70% ethanol at −20°C until DNA extractions. Glycogen and lipids from a homogenate of the remaining carcass were separated with a methanol–chloroform extraction. Lipids were quantified using a phospho-vanillin reaction of the supernatant compared with a standard curve of extra virgin olive oil (oleic acid) in chloroform (Vanhandel, 1985b) (54-μl olive oil in 50-ml chloroform; standard concentrations of 0, 1, 5, 10, 25, 50, 75, 100 μg), and the remaining pellet used in glycogen quantification with an anthrone assay compared with a standard curve of glycogen from oyster, type II (Sigma-Aldrich) (Vanhandel, 1985a) (25-mg oyster in 25-ml water; standard concentrations of 0, 1, 5, 10, 25, 50, 75, 100 μg). Protein assays were conducted following the Bio-Rad Bradford assay standard procedure for microtiter plates and calibration standards of bovine serum albumin (protein standard II, Bio-Rad Laboratories) (standard concentrations of 0, 25, 50, 100, 250 and 500 μg).

### Dietary breadth

#### DNA extraction and amplification

Total DNA from the alimentary canal was extracted using the DNeasy Blood and Tissue Kit (QIAgen GmbH, Hilden, Germany) following the manufacturer’s instructions and extracts recovered in a final volume of 400 μl. Because we were extracting DNA from pollen grains in the mid- and hindguts that had already undergone at least partial digestion by the bees, we reasoned that additional digests were not necessary. Dietary plant DNA from consumed nectar and pollen was amplified using a nested PCR procedure with primers targeting the *trn*L (UAA) p6 intron region of the chloroplast genome (Taberlet *et al.*, 1991, 2007). This approach amplified the small yet highly conserved gene region of interest, while simultaneously preparing individual samples for high-throughput sequencing by attaching sample-specific barcodes and sequencing oligonucleotides compatible with the Illumina MiSeq sequencing platform, as described by Hayden *et al.* (2008), Clarke *et al.* (2014) and Illumina (2013). This allowed for a cost-effective association of particular sequences to specific samples in a pooled DNA library (Pompanon *et al.*, 2012; Yu *et al.*, 2012).

The *trn*L primers were modified to include an overhanging adapter sequence (Table 2) such that sample-specific indexing primers would anneal to the first PCR product. In Phase 1 of the PCR, the amplification mixture included 8-μl PCR-grade water, 12.5 μl of KAPA HiFi HotStart ReadyMix 2x (KAPA Biosystems, Wilmington, MA), 1.0 μl each of 60 nmol modified forward and reverse *trn*L primers and 2.5 μl of template DNA, for a 25-μl total reaction volume. Samples were denatured at 95°C for 3 min, followed by 40 cycles of 30 sec at 98°C, 30 sec at 50°C and 30 sec at 72°C, with a final 3-min elongation at 72°C.

**Table 2:** *trn*L primers used in this study

**Primer**	**Sequence (5′-3′)**
Forward	*TCGTCGGCAGCGTCAGATGTGTATAAGAGACAG* **GGGCAATCCTGAGCCAA**
Reverse	*GTCTCGTGGGCTCGGAGATGTGTATAAGAGACAG* **CCATTGAGTCTCTGCACCTATC**

Italics indicate the additional adapter sequence, with the amplicon specific sequence in bold (Taberlet *et al.*, 1991, 2007).

To verify the presence of amplified DNA, samples were screened by electrophoresis on a 2% agarose gel. A total of 120 honey bees and 73 female *M. desponsa* specimens were evaluated for the presence of *trn*L, and only the positive samples underwent the second phase of amplification. This PCR mixture included 5.0 μl of PCR-grade water, 12.5 μl of KAPA HiFi HotStart ReadyMix 2x, 2.5 μl each of i5 and i7 Nextera XT indexing primers (Nextera XT index kit v2, Illumina Inc., San Diego, CA) (Supplemental Table 1) and 2.5 μl of *trn*L amplicon DNA from the first PCR. Samples were denatured at 95°C for 3 min, followed by 10 cycles of 30 sec at 98°C, 30 sec at 65°C and 30 sec at 72°C, with a final 3-min elongation at 72°C. Plant DNA from alfalfa, blue lupine, clover, dandelion, hairy vetch and a prairie seed mix were used as positive controls.

#### Library cleanup and sequencing

The library was cleaned using a 1.4:1.0 v:v ratio of AMPure XP beads (Agencourt Biosciences, Beverly, MA) to sample to remove fragments <150 bp (Lennon *et al.*, 2010). Bead-bound DNA was eluted in 1× TE buffer and then quantified using the Quant-iT PicoGreen dsDNA assay kit (Invitrogen, Carlsbad, CA). Sequencing was done at the University of Illinois Biotechnology Center (Urbana, IL) using an Illumina MiSeq v2 platform 2x250 paired-end run. There were on average 3385 ± 23 raw data reads generated per sample. Because DNA from individual bee samples had unique indexing primers, 384 samples were combined and sequenced in a single library.

#### Sequence analysis

Sample de-multiplexing and quality filtering were performed using a modified OBITools pipeline (http://metabarcoding.org/obitools/) (Boyer *et al.*, 2016). This program was developed specifically for the analysis of next-generation sequencing data in a metabarcoding context, which is particularly relevant for dietary breadth analysis. This was installed and run within QIIME v.1.9.1 VirtualBox (http://qiime.org/install/virtual_box.html). Full-length amplicon sequences were first recovered by aligning the forward and reverse reads (illuminapairedend command) and only well-recovered amplicon sequences were kept (obigrep command, minimum alignment score of 20). The primer sequences were identified and removed and the barcode sequence was reverse complemented if needed (ngsfilter command). Barcodes were then dereplicated (obiuniq command). We selected sequences longer than 20 that occurred at least 10 times for the assignment of operational taxonomic units (OTUs) (obigrep command), as *trn*L (UAA) p6 intron region ranges from 10 to 143 bp (Taberlet *et al.*, 2007). A reference database of North American *trn*L sequences was downloaded from the NCBI database and sequences assigned to taxa with 90% sequence similarity (obiconvert and ecotag commands).

Raw sequence reads are deposited and publicly available in the Sequence Read Archive (https://www.ncbi.nlm.nih.gov/sra) (BioProject ID: PRJNA558996; accession numbers: SAMN12510563 and SAMN12510732).

### Statistical analyses

#### Landscape composition

The 2013 Cropland Data Layer (USDA, National Agricultural Statistics Service; https://nassgeodata.gmu.edu/CropScape/) land-use data were imported into ArcMap 10.3.1 (ESRI, 2015). The land cover was extracted by mask in 3-km buffers around each site, approximating the foraging range of honey bees and larger native pollinators (Cariveau *et al.*, 2013). A raster file of land use was exported in a GRID format and imported into FRAGSTATS v.4.2.1 (McGarigal *et al.*, 2012). A full landscape metrics analysis was run and included number of patches, largest patch index (LPI), total edges, landscape shape index, mean patch area (AREA_MN), radius of gyration distribution (GYRATE_AM), fractal dimension index, perimeter-area ratio, contiguity index, core area index, Euclidian nearest neighbour distance and relative patch richness. See Supplemental Table 2 for variable units and definitions.

#### Landscape and bee macronutrients

Multiple regression analysis was used in SAS (SAS Institute Inc, Cary, North Carolina, USA) to investigate relationships of the macronutrient variables (glycogen, lipids and total protein) between bee species. Because the landscape explanatory variables are site-level variables, the regression analysis was done at the site level (*n* = 12). To accomplish this, the three macronutrient variables were averaged within each site per bee species, resulting in six response variables. Because the response within each site was multivariate in nature, in order to compare bee species, the modelled response variable for each health metric at a given site was calculated as the difference in the means of *M. desponsa* and honey bees within that site. Analysing each bee species in separate regressions would not have allowed for direct comparisons between their macronutrient responses to changing landscape conditions. Stepwise selection was used to select explanatory landscape variables for these models, with significance of 0.15 to enter and remain in the model.

#### Dietary composition and bee macronutrients

Linear mixed model regression analysis was used to model the macronutrients (glycogen, lipids and protein) as functions of dietary composition. For each of the bee species, separate models were developed for the macronutrients, and when residual analysis indicated a violation of the normality assumptions, the natural log of the macronutrient was used as a response in the model. This occurred for glycogen for both bee species and lipids for honey bees. Dietary composition variables were identified for each taxon from the gut-content analysis and initially included 51 OTUs. In order to reduce the number of dietary variables, each bee species and macronutrient combination was subjected to an initial stepwise regression to identify the most significant plant resources, with a significance level of 0.15 used to enter and remain in the model.

Regression models were fit using fixed effects for the dietary composition variables and a random effect for site (*n* = 12), resulting in linear mixed regression models. The random effect for site was included in order to account for potential variation from one site to another. Influence analysis was performed for each model to identify observations having unusual influence on the model, in conjunction with outlier identification.

Significant differences between bee species for dietary preferences, as measured by the number of reads from the family-level plant classification (OTU) (*n* = 9), were tested within each of the 12 sites using a chi-square test of homogeneity, with post hoc pairwise comparisons for significant overall treatments using a Bonferroni correction. Family-level analyses were conducted as some plant taxa were only represented in a portion of the samples from some locations, and consolidating OTUs into families ensured adequate comparisons between bee species at each sampling location.

## Results

### Landscape and bee macronutrients

**Figure 2:**
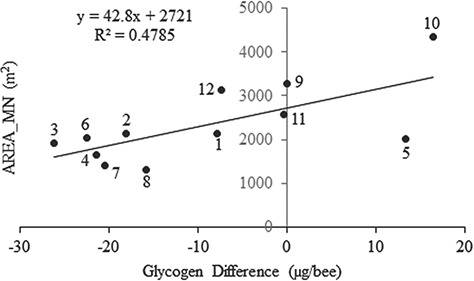
The relationship between mean habitat patch area and glycogen levels, expressed as the difference between *M. desponsa* and honey bees, per sampling site. Points are labelled with sampling locations.

Stepwise selection revealed that the AREA_MN was the significant landscape predictor of the glycogen differences observed between *M. desponsa* and honey bees across sampling sites ($\hat{\beta}$=10.1; F = 9.17, df = 1,10, *P* = 0.013). As the mean area of the patch size increased, so did the glycogen difference between *M. desponsa* and honey bees, indicating that uniformly larger habitat patches in the landscape were beneficial to glycogen levels in honey bees, while *M. desponsa* had relatively higher glycogen levels in areas with smaller habitat patches (Fig. 2). This could also imply that larger patches were dominated by honey bees, while *M. desponsa* were confined to relatively smaller habitat patches.

**Figure 3:**
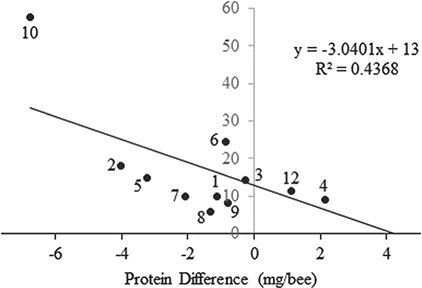
The relationship between largest patch index and protein levels, expressed as the difference between *M. desponsa* and honey bees, per sampling site. Points are labelled with sampling locations.

Protein differences were significantly influenced by the LPI, a measure of single patch dominance in the landscape ($\hat{\beta}$= − 0.50; F = 8.26, df = 2,9, *P* = 0.018) and GYRATE_AM, which measures how far an organism can move from a random starting point in a random direction without leaving a patch ($\hat{\beta}$=0.024; F = 4.51, df = 2,9, *P* = 0.063), though the parameter estimate for this second variable indicates the magnitude of this effect is small. The negative relationship between LPI and protein indicates that larger dominant patches reduce protein levels in honey bees relative to *M. desponsa* (Fig. 3).

**Figure 4:**
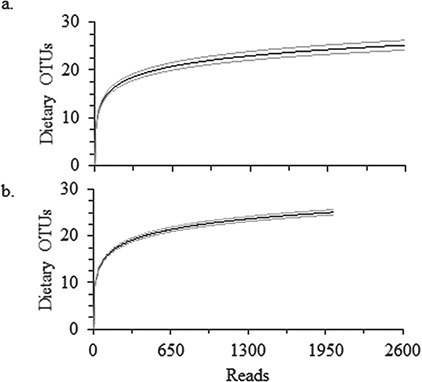
Alpha rarefaction curves of the filtered sequence reads showing the number of dietary plant OTUs, including Poaceae, recovered per sampling depth for honey bees (a) and *Melissodes desponsa* (b). Gray lines indicate the SE of the mean.

None of the measured landscape variables explained differences in lipids between sampling locations. The glycogen, lipid and protein results for each of the bee species and collection site are shown in Table 2, as well as Supplemental Figs 1–3.

### Dietary composition and bee macronutrients

The data for individual bee macronutrient and dietary results are presented in the supplemental spreadsheet. After quality filtering, there were a total of 221 842 sequence reads, from which 60.4% (134026) came from *trn*L positive honey bee samples (*n* = 103) and 39.6% (87 816) came from *M. desponsa* (*n* = 67). Figure 4 shows the results of rarefaction analysis, indicating that the sampling depth was sufficient to capture the diversity of plant species in the bees’ diets in the study region. A total of 51 OTUs were identified to species (10), genus (15) or family (22), with the remaining OTUs (4) determined to be unique but not matching known *trn*L sequences from the NCBI database. Recovered OTUs represent plant species from at least 18 different families. Individual honey bee samples ranged from 4–34 plant OTUs and *M. desponsa* ranged from 17–31 plant OTUs.

Poaceae accounted for 78% of sequence reads across all bee samples, which was attributed to forage contamination in the field. Though corn (*Zea mays*) is the dominant grass in the region and honey bees are frequently observed collecting corn pollen, this crop was not in anthesis during bee collection. Honey bees were not observed collecting pollen from other wild grasses, and as Poaceae was not present in controls, the abundance of this plant family in samples may have been due to possible preferential amplification of *trn*L during PCRs versus other plant species (Nichols *et al.*, 2018). Thus, this plant group was excluded from downstream analyses to prevent skewness in results.

Residual analysis of macronutrients and dietary composition regression analyses indicated non-normality of glycogen for both bee species and lipids for honey bees—the glycogen and lipid responses were therefore log transformed, which resulted in model residuals that conformed more closely to normality assumptions. The remaining models were run using untransformed data, and model results are presented in Supplemental Tables 3–8. While significant, the magnitude of the effects of individual plant OTUs on overall patterns of glycogen, lipid and protein levels were subtle regardless of their overall significance in individual models, as evidenced by standardized coefficient values.

**Figure 5:**
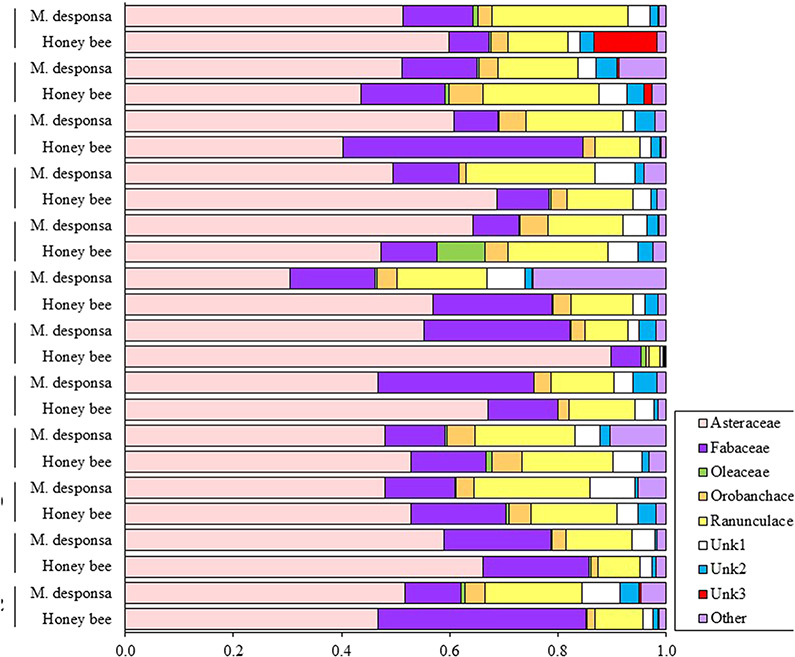
Average proportion of reads amplified for the most abundant dietary plant families, by bee species and site. Families representing <0.5% of reads were grouped as "other."

The proportion of sequence reads from each site for each bee species for the most common flowering families are shown in Fig. 5, which were significantly different between bee species within sites (Table 3). Between sites there was dietary overlap (Table 4); however, *M. desponsa* foraged more frequently on Ranunculaceae, Orobanchaceae and less common flowering species (Other) compared with honey bees (Table 5). Both bees foraged on flowers of Asteraceae, Fabaceae and Oleaceae. When honey bees foraged more heavily on Asteraceae than *M. desponsa* (sites 5, 6, 7 and 9; Table 5), this was explained by preferential foraging on species Asteraceae1 and Asteraceae7 specifically. When honey bees foraged more heavily on Fabaceae (sites 1, 7, 10; Table 5), this was explained by preferential foraging on *Medicago* sp., *Melilotus* sp. and Fabaceae1. *Melissodes desponsa* also foraged heavily on these species, particularly at sites 5, 6 and 12, in addition to *Trifolium* sp.

**Table 3:** Results of χ^2^ test of homogeneity for bee dietary composition within sites

**Site**	**χ^2^**	**d.f.^1^**	***P***
1	450	8	<0.001
2	30.0	7	<0.001
3	81.9	8	<0.001
4	78.0	7	<0.001
5	250	7	<0.001
6	1250	8	<0.001
7	684	8	<0.001
8	168	8	<0.001
9	70.8	7	<0.001
10	433	8	<0.001
11	132	8	<0.001
12	119	8	<0.001

^1^Bees from some sites did not contain DNA from Unk3 and thus that category was excluded for those analyses.

**Table 4:** Dietary composition by pollinator species

OTU	Honey bee (*n* = 103)			*M. desponsa* (n = 67)		
	Sequence reads	Proportion (%)^a^	Present in samples	Sequence reads	Proportion (%)	Present in samples
Apiaceae	28	0.021	23	11	0.013	8
Apocynaceae	131	0.098	59	62	0.071	43
Asteraceae1	17 669	13.2	102	5741	6.54	67
Asteraceae2	714	0.533	94	461	0.525	60
Asteraceae3	4	0.003	3	1055	1.20	17
Asteraceae4	131	0.098	63	96	0.109	46
Asteraceae5	332	0.248	74	105	0.120	45
Asteraceae6	324	0.242	12	0	0	0
Asteraceae7	531	0.396	96	175	0.199	57
Asteraceae:
*Antennaria dioica*	9	0.007	8	0	0	0
*Eupatorium cannabinum*	23	0.017	16	15	0.017	9
*Pilosella officinarum*	8	0.006	7	5	0.006	4
*Solidago virgaurea*	46	0.034	15	5	0.006	3
Boraginaceae	37	0.028	28	21	0.024	17
Brassicaceae	79	0.059	47	50	0.057	33
Convolvulaceae	20	0.015	16	35	0.040	27
Curcubitaceae: *Curcubita pepo*	1	0.001	1	187	0.213	7
Cyperaceae: *Carex*	13	0.010	13	9	0.010	9
Fabaceae1	1247	0.931	27	21	0.024	6
Fabaceae2	692	0.516	102	381	0.434	65
Fabaceae3	44	0.033	32	117	0.133	26
Fabaceae4	10	0.007	5	0	0	0
Fabaceae:
*Amorpha fruticosa*	101	0.075	54	55	0.063	33
*Glycine max*	479	0.357	62	318	0.362	49
*Medicago* sp.	2605	1.94	98	637	0.725	66
*Melilotus* sp.	751	0.560	78	714	0.813	44
*Trifolium* sp.	218	0.163	25	105	0.120	10
Hypoxidaceae	58	0.043	32	47	0.054	25
Lamiaceae:
*Nepeta* sp.	0	0	0	39	0.044	5
*Salvia* sp.	26	0.019	20	10	0.011	9
Malvaceae1	0	0	0	133	0.151	2
Malvaceae2	1	0.001	1	108	0.123	8

**Table 4:** Continued

OTU	Honey bee (*n* = 103)			*M. desponsa* (n = 67)		
	Sequence reads	Proportion (%)^a^	Present in samples	Sequence reads	Proportion (%)	Present in samples
Oleaceae	297	0.223	41	53	0.060	35
Orobanchaceae: *Pedicularis*	848	0.633	93	528	0.601	67
Poaceae1	69 161	51.6	102	51 345	58.5	67
Poaceae:
*Bouteloua* sp.	439	0.328	92	242	0.276	59
*Bromus* sp.	22 110	16.5	97	15 145	17.2	67
*Cenchrus* sp.	381	0.284	84	268	0.305	65
*Melinis repens*	70	0.052	47	49	0.056	33
*Muhlenbergia* sp.	235	0.175	78	172	0.196	60
*Panicum notatum*	54	0.040	38	37	0.042	26
*Paspalum* sp.	66	0.049	42	39	0.044	33
*Sorghastrum nutans*	8757	6.53	97	5711	6.50	67
Polygonaceae: *Persicaria*	86	0.064	56	80	0.091	42
Ranunculaceae:
*Thalictrum* sp. 1	2349	1.75	95	1600	1.82	66
*Thalictrum* sp. 2	1062	0.792	92	615	0.700	64
Solanaceae	1	0.001	1	112	0.128	2
Unknown1	865	0.645	87	613	0.698	66
Unknown2	493	0.368	78	422	0.480	50
Unknown3	319	0.238	15	18	0.020	8
Unknown4	88	0.066	41	62	0.071	34

^a^Proportion refers to the percentage of total sequence reads from each pollen.

**Table 5:** Results of pairwise χ^2^ tests of homogeneity for bee diet within sites

Site	Asteraceae^a^^,^^b^	Fabaceae	Oleaceae	Orobanchaceae	Ranunculaceae	Unk1	Unk2	Unk3	Other
1	3.28 0.039	***155*** ***<0.001***	*8.08* *0.001*	*15.1* *<0.001*	*47.8* *<0.001*	*57.2* *<0.001*	*25.7* *<0.001*	0.443 0.449	*34.7* *<0.001*
2	3.14 0.037	0.002 0.956	0.003 0.947	3.58 0.026	*8.61* *<0.001*	5.26 0.007	1.13 0.212	0.015 0.885
3	1.81 0.12	5.26 0.007	0.310 0.515	0.710 0.325	*6.68* *0.003*	*15.3* *<0.001*	***11.9*** ***<0.001***	0.269 0.544	*17.6* *<0.001*
4	1.91 0.066	2.55 0.034	2.19 0.049	0.205 0.547	0.590 0.307	0.473 0.360	1.25 0.138	*34.9* *<0.001*
5	***40.3*** ***<0.001***	*83.7* *<0.001*	0.239 0.557	2.77 0.045	0.166 0.624	0.002 0.955	*45.3* *<0.001*	0.083 0.730
6	***163*** ***<0.001***	*439* *<0.001*	***6.25*** ***0.003***	*44.0* *<0.001*	*104* *<0.001*	*26.8* *<0.001*	*91.3* *<0.001*	1.63 0.135	*37.2* *<0.001*
7	***66.1*** ***<0.001***	***8.95*** ***0.001***	1.74 0.140	0.158 0.656	*10.5* *<0.001*	*36.1* *<0.001*	2.78 0.062	3.13 0.047	*416* *<0.001*
8	*26.7* *<0.001*	1.78 0.094	***63.6*** ***<0.001***	0.980 0.214	***6.65*** ***0.001***	0.994 0.211	1.20 0.169	2.20 0.063	2.69 0.039
9	***13.8*** ***<0.001***	1.63 0.178	0.733 0.365	2.79 0.078	*25.5* *<0.001*	*10.8* *0.001*	0.688 0.381	*7.52* *0.004*
10	*55.4* *<0.001*	***204*** ***<0.001***	*6.58* *0.004*	*13.8* *<0.001*	*52.2* *<0.001*	0.202 0.616	*9.95* *<0.001*	0.196 0.621	5.46 0.009
11	*2.11* *0.002*	0.439 0.150	0.076 0.550	***3.66*** ***<0.001***	***4.68*** ***<0.001***	***1.81*** ***0.004***	0.303 0.232	***5.54*** ***<0.001***	*9.31* *<0.001*
12	3.51 0.044	*10.1* *0.001*	2.43 0.094	0.223 0.612	*43.6* *<0.001*	4.25 0.027	1.74 0.157	***36.6*** ***<0.001***	0.308 0.551

^a^χ^2^ value on top, *P*-value on bottom; df = 1 for each.

^b^Bonferroni correction applied; therefore, italic values indicate significance at *P* < 0.006. Bold values indicate that the significance was driven by higher than expected values in honey bees, while regular italicized indicates significance driven by higher values in *M. desponsa*.

## Discussion

This study adds to our understanding of how a co-occurring native and managed bees may be physiologically impacted in relation to each other by landscape and dietary variability across their environment. Our data show that while the macronutrient ratios of these bees are significantly affected by certain landscape patch characteristics, albeit divergently, the dietary composition recovered from *trn*L did not affect physiology as measured by glycogen, lipids and protein. Significant differences in dietary preference within sampling sites, however, suggest that current management considerations favouring honey bee-oriented conservation plantings may be better served by augmenting vegetation in the environment preferred by native bees as well, which will benefit honey bees by proxy.

Physiological glycogen, lipid and protein levels in insects have been reported throughout the literature particularly as they relate to various stress responses. However, the value range that constitutes a healthy versus a deficient honey bee with regards to these cannot yet be defined due to variability in sampling methodology and the context of the experiments. Smart *et al.* (2019) reported glycogen, lipid and protein concentrations in honey bees as mg/g wet weight (we reported units as μg per individual bee) with the alimentary canal included in their analysis. They demonstrated a positive correlation between abdominal glycogen, lipids and proteins and frames of winter bees in the colonies, indicating that higher concentrations are associated with increased populations and survivorship within the colony. Mogren and Lundgren (2016) reported glycogen and lipid levels roughly two times greater in honey bees collected from pollinator strips adjacent to conventional and organic corn fields after the alimentary canal had been removed than in the present study. As more studies are published using these biomarkers in honey bees and other pollinators, comparisons will be possible that identify optimum macronutrient concentrations at various times during the year, corresponding with changes in colony physiology related to overwintering (Dolezal *et al.*, 2016), starvation stress (Wang *et al.*, 2016), pesticide exposures (Mogren and Lundgren, 2016; Cook, 2019) and pathogens(Lopienska-Biernat *et al.*, 2017). Presently, we are constrained to relative comparisons between locations and pollinator species.

From a landscape perspective, the solitary bee *M. desponsa* had higher glycogen and protein levels as compared with honey bees when there was patch dominance, as measured by LPI. In a previous study, Mogren *et al.* (2016) categorized land use types in eastern South Dakota, noting that 75% of the landscape comprised corn, soybeans and pasture, all of which represent highly simplified land-use designations. The same study found that these large patches of monocrops led to increased abundance of certain native bees, including *M. desponsa*, among others, contrary to what would be expected in a historic prairie region. These agrobiont pollinators appear to thrive in otherwise suboptimal habitat and increased glycogen and protein concentrations imply a physiological benefit resulting from some aspect of this otherwise degraded habitat that cannot be utilized by other bee species.

In contrast, honey bees experienced physiological benefits, relative to *M. desponsa*, in the form of increased glycogen and total protein concentrations when patch sizes were homogenous, which has also been shown to decrease pollen foraging distances (Steffan-Dewenter and Kuhn, 2003). This supports the management decisions of beekeepers in the region, who avoid corn and soybean fields when establishing apiaries (Otto *et al.*, 2016). Interestingly, we did not find an effect of landscape on lipid levels between bee species; elsewhere, increasing landscape diversity positively influenced bee lipid levels (Dolezal *et al.*, 2016; Smith *et al.*, 2016; Alaux *et al.*, 2017; Smart *et al.*, 2019). Lipids are synthesized and stored in the insect fat body as a source of energy; for honey bees, they are critically important to overwintering success and are higher in fall bees, with the temporal polyethism characterizing the species also contributing to lipid changes through time (Döke *et al.*, 2015). Solitary bees, such as *M. desponsa*, acquire all necessary nutrition themselves, as opposed to nest mates contributing to resource collection as in honey bees. In their case, lipids will be critical for egg production in females (Lawson *et al.*, 2017). We did not categorize the reproductive status of *M. desponsa* during dissections, though developed eggs in the ovaries were not apparent. Thus, a lack of significance in our lipids data may be attributed to a pre- or post-reproductive biological status of *M. desponsa* that does not reflect potential impacts of landscape diversity.

Dietary composition, as indicated by DNA recovered in gut-content analyses, had no effect on glycogen, lipid or protein levels for either bee species. In the bumble bee *Bombus terrestris*, Kämper *et al.* (2016) found that forage quantity was more important than diversity in colony growth, which supports our finding, at least for the social honey bees. In contrast, Alaux *et al.* (2017) found that the presence of flowering catch crops increased pollen diet diversity, in turn leading to greater fat body mass and vitellogenin levels in honey bees. Honey bee hypopharyngeal gland development and vitellogenin expression were positively influenced by the nutritive quality of pollen as opposed to pollen diversity (Di Pasquale *et al.*, 2016), though polyfloral diets may improve survival outcomes when honey bees are faced with a secondary stressor, such as the parasite *Nosema ceranae* (Di Pasquale *et al.*, 2013). A lack of an observed physiological response to dietary composition in the present study could be attributed to both bee species obtaining sufficient nutrition from the quantity and diversity of forage available in this simplified landscape in eastern South Dakota. Alternatively, conditions may have been suboptimal for both species across the study region. More research is needed to determine exact macronutrient values that indicate healthy versus stressed bees.

Sequencing throughput per sample was lower for our samples than has been reported elsewhere in the literature for *trn*L dietary analyses (Valentini *et al.*, 2009; Hibert *et al.*, 2013; Srivathsan *et al.*, 2015; Gebremedhin *et al.*, 2016), though these studies examined faecal matter from herbivores as opposed to partially digested pollen. Low read counts may have been the result of using a DNA extraction protocol that was not plant-specific. However, despite low reads, rarefaction curves indicate sufficient coverage in our samples (Fig. 4).

While overall dietary composition may not have contributed to physiological differences in honey bees and *M. desponsa*, there were some differences in foraging preferences between the species at different field sites. At sites where honey bees foraged heavily on Fabaceae, this was driven by a preference for non-native *Melilotus* sp. and *Medicago* sp. within the Fabaceae. These plant genera were identified as predominant floral resources in North Dakota as well (Smart *et al.*, 2016; Otto *et al.*, 2017). Similarly, honey bees in temperate regions have been found to utilize other agricultural weeds (Requier *et al.*, 2015). While *M. desponsa* also foraged on *Melilotus* and *Medicago*, they preferred native, albeit less abundant floral species, such as Ranunculaceae and Orobanchaceae when they were present at study sites.

Studies on the floral preferences of many native bees are limited, though *Melissodes* as a genus are typically regarded as composite specialists, whose foraging is not thought to compete with that of honey bees (Dickinson and McKone, 1991). Our data showed dietary overlap between these two bee species particularly within Asteraceae and Fabaceae, though *M. desponsa* foraged on native flowers when they were apparently available (Table 5). Macronutrient ratios or other variables not measured in this study may also shape foraging strategies (Vaudo *et al.*, 2016). Since we only evaluated the dietary compositions of two bee species in the region, in which 32 species of Apoidea were recently identified (Mogren *et al.*, 2016), a full understanding of whether niche partitioning or competition occurs between the native solitary bees in the region and honey bees is not possible based on our data alone.

Although it is relatively simple to categorize the composition of honey bee collected pollen and nectar inside a managed colony, understanding the feeding biology of solitary native bees is more difficult. Thus, the application of metabarcoding techniques is an important tool for understanding their ecology in conservation applications. Unfortunately, being unable to assign a specific name to sequences in all cases can limit its utility when devising specific management plans. This emphasizes the need for bioinventories that simultaneously collect and deposit plant DNA for multiple genetic markers in public repositories, particularly in underrepresented regions of conservation importance. This is especially true for *trn*L, which has proven useful in dietary studies of herbivores using partially digested plant DNA (Valentini *et al.*, 2009; Hibert *et al.*, 2013; Srivathsan *et al.*, 2015; Wallinger *et al.*, 2015; Gebremedhin *et al.*, 2016; Espunyes *et al.*, 2019). While we were able to distinguish differences between the diets of honey bees and *M. desponsa* using *trn*L, the taxonomic resolution to genus and family as opposed to species level identification may explain why we were unable to detect an effect on macronutrient levels across the study area. In some cases, we could distinguish OTUs despite not knowing species names (e.g. Asteraceae; Table 4), which still counted as dietary diversity, but in others, we were only able to resolve sequences at the family level. The limitations posed by our experimental design necessitated the use of a gene target that could identify DNA that had already undergone partial digestion, but future studies utilizing *trn*L for pollinator gut-content analysis may benefit from concurrent assembly of a DNA reference library for locally flowering species.

Our results imply that in highly developed agricultural landscapes such as eastern South Dakota, management practices focused on honey bee conservation that promote ubiquitous and potentially weedy flowering species may not completely capture the needs of native bees when honey bees are also present. Otto *et al.* (2017) similarly concluded that honey bees may not be appropriate umbrella species for all pollinators within the landscape. However, we did find significant dietary overlap between these two bee species, specifically among weedy flowers. Dietary overlap may explain why we did not find an effect of dietary diversity on individual bee nutrient metrics. However, we did find a diverging physiological response to landscape characteristics, implying these bee species respond differently to agricultural development and have different ecological patch requirements despite dietary overlap. For honey bees, greater glycogen in response to increasing AREA_MN may be due to an enhanced success in floral patch discovery (Donaldson-Matasci and Dornhaus, 2014) versus smaller patches, which may be more difficult to discover and recruit to. Future pollinator conservation and management efforts in the region should incorporate minimum patch areas in planting recommendations to ensure these conservation plantings capture dietary and spatial needs for target species.

## Supplementary Material

supplementary_files_coaa109
