# Supplementary material for: Diverging landscape impacts on macronutrient status despite overlapping diets in managed (Apis mellifera) and native (Melissodes desponsa) bees
Source: Conserv Physiol. 2020 Dec 15;8(1):coaa109. doi: 10.1093/conphys/coaa109 (PMC7745716; doi:10.1093/conphys/coaa109)
Supplement: supplementary_files_coaa109 [file supplementary_files_coaa109.zip › Supplemental Files.docx]

**Supplemental Table 1.** Illumina Nextera XT indexing primer sequences.

| **i7 Indices** | | | **i5 Indices** | |
| --- | --- | --- | --- | --- |
| **Nextera Index** | **Sequence** | **Reverse Complement** | **Nextera Index** | **Sequence** |
| N701 | TCGCCTTA | TAAGGCGA | S502 | CTCTCTAT |
| N702 | CTAGTACG | CGTACTAG | S503 | TATCCTCT |
| N703 | TTCTGCCT | AGGCAGAA | S505 | GTAAGGAG |
| N704 | GCTCAGGA | TCCTGAGC | S506 | ACTGCATA |
| N705 | AGGAGTCC | GGACTCCT | S507 | AAGGAGTA |
| N706 | CATGCCTA | TAGGCATG | S508 | CTAAGCCT |
| N707 | GTAGAGAG | CTCTCTAC | S510 | CGTCTAAT |
| N710 | CAGCCTCG | CGAGGCTG | S511 | TCTCTCCG |
| N711 | TGCCTCTT | AAGAGGCA | S513 | TCGACTAG |
| N712 | TCCTCTAC | GTAGAGGA | S515 | TTCTAGCT |
| N714 | TCATGAGC | GCTCATGA | S516 | CCTAGAGT |
| N715 | CCTGAGAT | ATCTCAGG | S517 | GCGTAAGA |
| N716 | TAGCGAGT | ACTCGCTA | S518 | CTATTAAG |
| N718 | GTAGCTCC | GGAGCTAC | S520 | AAGGCTAT |
| N719 | TACTACGC | GCGTAGTA | S521 | GAGCCTTA |
| N720 | AGGCTCCG | CGGAGCCT | S522 | TTATGCGA |
| N721 | GCAGCGTA | TACGCTGC |  |  |
| N722 | CTGCGCAT | ATGCGCAG |  |  |
| N723 | GAGCGCTA | TAGCGCTC |  |  |
| N724 | CGCTCAGT | ACTGAGCG |  |  |
| N726 | GTCTTAGG | CCTAAGAC |  |  |
| N727 | ACTGATCG | CGATCAGT |  |  |
| N728 | TAGCTGCA | TGCAGCTA |  |  |
| N729 | GACGTCGA | TCGACGTC |  |  |

**Supplemental Table 2.** Summary of the landscape variable metrics included in the multiple regression analysis of bee macronutrient metrics.

| Site | NP^1^ | LPI | TE | LSI | AREA_MN | GYRATE_AM | FRAC_MN | PARA_MN | CONTIG_MN | CAI_MN | ENN_MN | RPR |
| --- | --- | --- | --- | --- | --- | --- | --- | --- | --- | --- | --- | --- |
| 1 | 1191 | 9.98 | 11,817 | 17.8 | 2,133 | 12.9 | 1.0451 | 1053 | 0.187 | 4.37 | 138 | 60.7 |
| 2 | 1190 | 18.1 | 11,051 | 16.7 | 2,142 | 15.5 | 1.0375 | 1066 | 0.174 | 4.08 | 138 | 82.1 |
| 3 | 1324 | 14.2 | 12,957 | 19.4 | 1,926 | 13.5 | 1.0416 | 1067 | 0.174 | 3.56 | 140 | 67.9 |
| 4 | 1538 | 9.09 | 14,729 | 21.9 | 1,656 | 10.3 | 1.0408 | 1057 | 0.182 | 4.07 | 126 | 60.7 |
| 5 | 1271 | 14.8 | 11,751 | 17.7 | 2,007 | 12.6 | 1.0418 | 1066 | 0.176 | 4.12 | 133 | 60.7 |
| 6 | 1248 | 24.6 | 12,367 | 18.5 | 2,043 | 17.4 | 1.0412 | 1048 | 0.189 | 4.65 | 134 | 64.3 |
| 7 | 1786 | 9.83 | 14,598 | 21.7 | 1,422 | 11.4 | 1.0404 | 1069 | 0.172 | 3.03 | 118 | 64.3 |
| 8 | 1952 | 5.82 | 16,154 | 23.9 | 1,305 | 7.95 | 1.0417 | 1062 | 0.177 | 3.45 | 122 | 64.3 |
| 9 | 774 | 8.35 | 8,249 | 12.7 | 3,285 | 11.3 | 1.0357 | 1037 | 0.193 | 6.60 | 185 | 64.3 |
| 10 | 587 | 57.7 | 6,949 | 10.9 | 4,338 | 34.3 | 1.0352 | 1079 | 0.167 | 4.54 | 193 | 60.7 |
| 11 | 986 | 8.65 | 9,886 | 15.1 | 2,583 | 14.6 | 1.0389 | 1028 | 0.195 | 4.68 | 145 | 71.4 |
| 12 | 814 | 11.3 | 8,222 | 12.7 | 3,123 | 15.7 | 1.0380 | 1046 | 0.187 | 4.91 | 176 | 60.7 |

^1^NP - Number of patches (total number of patches within the 3 km sampling radius); LPI – Largest patch index (percentage of the sampling region comprised by the largest patch); TE – Total edge (sum of the lengths in meters of all edges from abutting patches); LSI – Landscape shape index (a measure of geometric complexity of the landscape, measuring the perimeter-to-area ratio of the sampling region); AREA_MN – Mean patch area (m^2^); GYRATE_AM – Area-weighted mean of patch radius of gyration (measure of landscape continuity, defined as average distance (m) an organism can move from a random starting point in a random direction without leaving a patch); FRAC_MN – Mean patch fractal dimension (perimeter-area calculation describing degree of complexity of planar shapes, units 1-2); PARA_MN – Perimeter-area ratio distribution (measure of shape complexity equal to the ratio of the patch perimeter (m) to area (m^2^)); CONTIG_MN – Contiguity index distribution (assesses the spatial connectedness of cells within a grid-cell patch to provide an index (0-1) of patch boundary configuration); CAI_MN – Core-area index (percentage of the patch comprised of core area, or an edge to interior ratio); ENN_MN – Euclidean nearest neighbor distance (measure of patch isolation, shortest strait line distance (m) between a focal patch and its nearest neighbor of the same class from the patch center); RPR – Relative patch richness (patch richness within the sampling region as a percentage of the maximum potential richness)

**Supplemental Table 3.** Regression model of log(glycogen) for honey bees.

| **Variable** | **Parameter Estimate^a^** | **Standardized Coefficient** |
| --- | --- | --- |
| Intercept | 3.70*** | -4x10^-15^ |
| Lamiaceae: *Salvia* sp. | -0.147* | -0.130 |
| Solanaceae | 1.01* | 0.147 |
| Asteraceae6 | 0.004* | 0.151 |
| *Pilosella officinarum* | 0.352* | 0.158 |
| *Curcubita pepo* | 3.19*** | 0.465 |
| Asteraceae1 | -0.001*** | -0.525 |
| Asteraceae7 | 0.046** | 0.713 |
| Oleaceae | 0.027** | 0.733 |
| Asteraceae3 | -2.07*** | -0.733 |
| *Solidago virgaurea* | -0.224** | -0.864 |

^a^P<0.15*, P<0.05**, P<0.01***

**Supplemental Table 4.** Regression model of log(glycogen) for *M. desponsa*.

| **Variable** | **Parameter Estimate^a^** | **Standardized Coefficient** |
| --- | --- | --- |
| Intercept | 3.42*** | 1.14x10^-15^ |
| *Eupatorium cannabinum* | -0.175* | -0.210 |
| Fabaceae: *Melilotus* sp. | -0.003** | -0.220 |
| Orobanchaceae: *Pedicularis* sp. | -0.027** | -0.292 |
| Apiaceae | -0.321*** | -0.292 |
| Cyperaceae: *Carex* sp. | -0.479** | -0.294 |
| Lamiaceae: *Salvia* sp. | 0.418*** | 0.298 |
| Unknown1 | -0.027** | -0.324 |
| Fabaceae1 | 0.055** | 0.375 |
| Unknown4 | 0.178*** | 0.396 |
| *Solidago virgaurea* | 0.708** | 0.505 |
| Asteraceae2 | 0.026** | 0.560 |
| Fabaceae: *Trifolium* sp. | -0.055*** | -1.07 |

^a^P<0.15*, P<0.05**, P<0.01***

**Supplemental Table 5.** Regression model of log(lipids) for honey bees.

| **Variable** | **Parameter Estimate^a^** | **Standardized Coefficient** |
| --- | --- | --- |
| Intercept | 3.66*** | 0.012 |
| Fabaceae: *Melilotus* sp. | 0.007* | 0.135 |
| Fabaceae: *Trifolium* sp. | 0.009* | 0.146 |
| Boraginaceae | -0.143* | -0.148 |
| Unknown3 | -0.007* | -0.160 |
| Brassicaceae | -0.107* | -0.167 |
| Unknown2 | 0.022* | 0.168 |
| Hypoxidaceae | 0.143*** | 0.234 |

^a^P<0.15*, P<0.05**, P<0.01***

**Supplemental Table 6.** Regression model of lipids for *M. desponsa*.

| **Variable** | **Parameter Estimate^a^** | **Standardized Coefficient** |
| --- | --- | --- |
| Intercept | 64.0*** | 1.63x10^-17^ |
| Malvaceae2 | 1.37* | 0.193 |
| Polygonaceae: *Persicaria* sp. | 8.87** | 0.268 |
| *Eupatorium cannabinum* | -19.1** | -0.278 |
| Asteraceae5 | 9.15** | 0.033 |
| Asteraceae1 | -0.297*** | -0.522 |
| Unknown2 | 2.90*** | 0.605 |

^a^P<0.15*, P<0.05**, P<0.01***

**Supplemental Table 7.** Regression model of protein for honey bees.

| **Variable** | **Parameter Estimate^a^** | **Standardized Coefficient** |
| --- | --- | --- |
| Intercept | 11.4*** | 2.67x10^-16^ |
| Lamiaceae: *Salvia* sp. | -1.08* | -0.142 |
| *Amorpha fruticosa* | 0.530* | 0.159 |
| *Antennaria dioica* | 3.18** | 0.222 |
| Asteraceae4 | 0.728** | 0.246 |
| Orobanchaceae: *Pedicularis* sp. | -0.211*** | -0.340 |

^a^P<0.15*, P<0.05**, P<0.01***

**Supplemental Table 8.** Regression model of protein for *M. desponsa*.

| **Variable** | **Parameter Estimate^a^** | **Standardized Coefficient** |
| --- | --- | --- |
| Intercept | 9.65*** | -0.020 |
| Convolvulaceae | 0.985* | 0.177 |
| Malvaceae2 | 0.132* | 0.210 |
| Oleaceae | 0.946** | 0.227 |
| *Amorpha fruticosa* | 0.951** | 0.257 |
| Asteraceae1 | -0.018* | -0.357 |
| Asteraceae7 | -0.556*** | -0.444 |
| Asteraceae5 | 1.19*** | 0.571 |

^a^P<0.15*, P<0.05**, P<0.01***

**Supplemental Figure 1.** Average glycogen per site ± SE for honey bees and *M. desponsa*.

**Supplemental Figure 2.** Average lipids per site ± SE for honey bees and *M. desponsa*.

**Supplemental Figure 3.** Average protein per site ± SE for honey bees and *M. desponsa*.
